# Environmental drivers defining linkages among life-history traits: mechanistic insights from a semiterrestrial amphipod subjected to macroscale gradients

**DOI:** 10.1002/ece3.759

**Published:** 2013-09-17

**Authors:** Julio Gómez, Francisco R Barboza, Omar Defeo

**Affiliations:** UNDECIMAR, Departamento de Ecología y Evolución, Facultad de Ciencias, UdelaRIguá 4225, Montevideo, Uruguay

**Keywords:** Environmental gradients, functional linkages, life-history traits, mixed models, phenotypic variability, Talitridae

## Abstract

Determining the existence of interconnected responses among life-history traits and identifying underlying environmental drivers are recognized as key goals for understanding the basis of phenotypic variability. We studied potentially interconnected responses among senescence, fecundity, embryos size, weight of brooding females, size at maturity and sex ratio in a semiterrestrial amphipod affected by macroscale gradients in beach morphodynamics and salinity. To this end, multiple modelling processes based on generalized additive mixed models were used to deal with the spatio-temporal structure of the data obtained at 10 beaches during 22 months. Salinity was the only nexus among life-history traits, suggesting that this physiological stressor influences the energy balance of organisms. Different salinity scenarios determined shifts in the weight of brooding females and size at maturity, having consequences in the number and size of embryos which in turn affected sex determination and sex ratio at the population level. Our work highlights the importance of analysing field data to find the variables and potential mechanisms that define concerted responses among traits, therefore defining life-history strategies.

## Introduction

Environment as an ecological actor causes variations in phenotype (phenotypic variability) and therefore in the performance of individuals (Pigliucci 2001). The physiological interdependence among life-history traits constrains and adjusts plastic responses, determining that the effects of a certain environmental pressure on one trait, has an effect on the others (Ricklefs and Wikelski 2002). Therefore, elucidating which variables are responsible for the observed phenotypic variability and the linkages among traits has been recognized as a key goal for understanding the basis of phenotypic variability in natural populations (Ricklefs and Wikelski 2002; Isaksson et al. 2011).

Widely distributed species that are subject to a broad range of conditions represent useful frameworks for evaluating environmental effects on phenotypic variability (Joshi et al. 2001; Defeo and McLachlan 2005). Water-land transitions in the evolutionary history of the talitrid amphipods (Hurley 1968; Spicer et al. 1987), the variety of habitats in which they live and the large geographical distribution that they exhibit (Ramus and Forward 2012), made them key cases for this kind of study. Particularly, those that inhabit sandy beaches evolved to persist in this harsh environment, where life histories are considered to be mainly shaped by physical factors (Defeo and McLachlan 2005). In this context, a morphodynamic spectrum from narrow and steep beaches (reflective beaches) to wide and flat ones (dissipative beaches) (Short 1999) affects sandy beach macrofauna, including amphipods (Defeo and Gómez 2005; McLachlan and Brown 2006). In addition, talitrid amphipods are affected by desiccation and osmotic constrains (Truchot 1990; Morritt and Spicer 1998), as they are exposed to semiterrestrial conditions and salinity fluctuations (Gómez and Defeo 2012; Ramus and Forward 2012).

**Figure 1:**
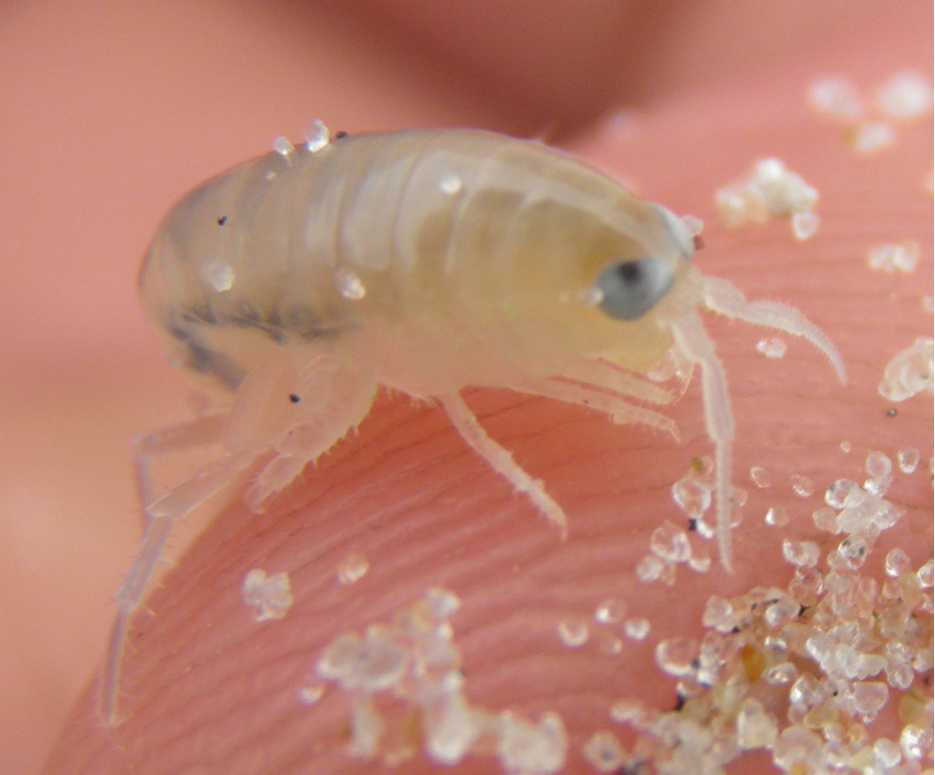
The talitrid amphipod *Altantorchestoidea brasiliensis*.

Uruguayan sandy beaches cover a wide morphodynamic range along the Rio de la Plata estuary (the widest worldwide) and the Atlantic Ocean, providing an ideal scenario to study how the environment affects life-history traits. This is particularly true in the talitrid amphipod *Altantorchestoidea brasiliensis* (Fig. 1), which inhabits the whole morphodynamic spectrum and, at the same time, is affected by the estuarine gradient generated by the Rio de la Plata. We used field data of this talitrid amphipod and supervised machine learning methods with mixed models to elucidate the environmental drivers that affect the response of multiple life-history traits and to understand the involved mechanisms.

## Material and Methods

### Study area and field sampling

The Uruguayan coastline stretches over 320 km along the north-eastern bank of the Rio de la Plata estuary and 210 km of adjacent Atlantic coastline (Lercari and Defeo 2006). Ten sandy beaches were sampled along this temperate microtidal coast, covering a wide range of morphodynamic states along the estuarine gradient. Environmental and biological samples were obtained every 2 months from July 1999 to April 2001. Three transects were set up perpendicular to the shoreline and spaced 8 m apart. On each transect, sampling stations were carried out every 4 m from the base of the dunes to the seaward limit of the *A. brasiliensis* distribution, until at least two consecutive stations yielded no amphipods. At each station, a sheet metal cylinder (27 cm diameter) was used to remove the sediment up to a depth of 40 cm. Samples were field-sieved through a 0.5 mm mesh; samples with coarse sand were carefully sorted in the laboratory to retain all organisms. At each sampling station, sediment samples were collected with a corer (6.5 cm diameter) for determination of mean grain size, sand moisture and organic matter content. Measurements of sediment temperature and sand compaction were made, in the latter using a piston pocket penetrometer (Herrick and Jones 2002). Salinity and water temperature were measured. Wave height and wave period were determined visually using a stopwatch. Beach slope was estimated by Emery's profiling technique (Emery 1961). Beach width was measured as the distance between the base of the dunes and the lower swash level.

In the laboratory, collected amphipods were measured from the tip of the cephalon to the end of the telson, using binocular microscopes equipped with micrometrical ocular lens, calibrated with objective micrometres. Individuals were sexed based on the presence of copulatory appendages in males, and the presence of oostegites, with or without setae, in females (Marques et al. 2003). Females with setae in the oostegites were considered as mature, as they had, have or will have embryos. We consider as brooding females only those carrying embryos. Individuals with no secondary sexual dimorphic features were classified as juveniles. Embryos were separated from the brood pouch, counted and measured. Afterwards, all individuals were dried at 50°C for 24 h and weighted to the nearest 0.0001 g. We estimated abundance (individuals per strip transect; ind·m^−1^) (Brazeiro and Defeo 1996) discriminated by size class for males, females and the whole population (adults and juveniles). Eleven size classes of 1.00 mm (from ≥2.50 to <13.50 mm) were considered.

### Modelling process

We modelled abundance by size class, number of embryos per female, brooding females dry weight, embryos size, proportion of mature females and proportion of females, in relation to environmental variables, using generalized additive mixed models (GAMM) (see details in Table 1). To do so, the gamm4 R package was used (Wood 2011). A categorical site variable and the date of each sampling event were included as random intercepts in all models, to adjust the variability among sites and times without consuming a great amount of degrees of freedom. All potential predictors were included without theoretical constrains, being the parsimony of the models (evaluated using Akaike's Information Criterion), the only basis to retain them. Variables were considered as smooth terms using penalized regression splines with degrees of freedom up to 3 (Hastie and Tibshirani 1990; Barboza et al. 2012; Gómez and Defeo 2012). Residuals were checked for all models.

**Table 1:** Details of Generalized Additive Mixed Models used for modelling life-history traits of *Altantorchestoidea brasiliensis*. In each model, all potential predictors were included without theoretical constrains; being the parsimony of the models, the only basis to retain them.

Response variable	Distribution	Link function	Trait related to the model
Abundance of males^1^	Gaussian	Identity	Males senescence
Abundance of females^1^	Gaussian	Identity	Females senescence
Total abundance^1^	Gaussian	Identity	Population senescence
Number of embryos per female	Poisson	Log	Fecundity
Brooding females dry weight	Gaussian	Identity	Energy availability
Embryos size	Gaussian	Identity	Embryos size
Proportion of mature females	Binomial	Logit	Size at maturity
Proportion of females	Binomial	Logit	Sex ratio

1 Abundance was calculated by size class and log-transformed.

## Results and Discussion

**Figure 2:**
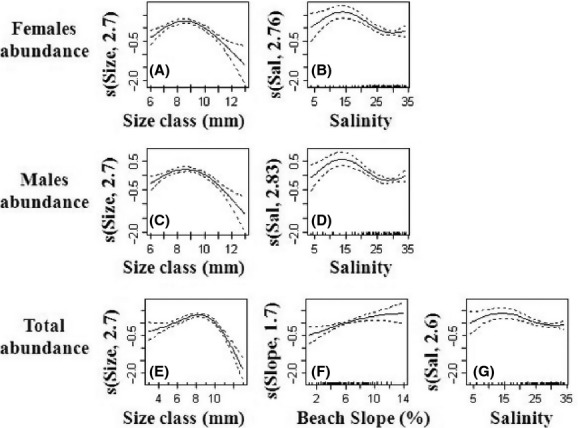
Gaussian GAMM of abundance (log-transformed) by size class: females (A, B), males (C, D) and total population (adults and juveniles) (E, F, G). Partial effects of retained predictors (presented in order of importance) are shown. The numbers in the *y*-axis labels indicate the estimated degrees of freedom of the smooth curve. Marks on the *x*-axis indicate the measured values of each variable.

Abundance models for males and females retained size class, and salinity as the best explanatory predictors of the observed variability (Fig. 2A–D). The partial response of log-abundance in relation to size class (considered as a proxy of age, see Gómez and Defeo 1999) showed that senescence (as a constant decay) in *A. brasiliensis* starts at ca. 9 mm both for males and females (Fig. 2A, C). In the particular case of females, this result coincides with the size at which 50% are in a brooding condition according to the observations of Gómez and Defeo 1999 and the results for female's maturity obtained herein (Fig. 4A). This suggests that *A. brasiliensis* females might be reproducing only once in this temperate region, increasing its mortality as a consequence of the large physiological energy invested in reproduction (“reproductive senescence”, see Kirkwood and Austad 2000). Salinity was the single and main environmental predictor of adult abundance. Both sexes exhibited their highest abundance at salinities near 15, consistently with laboratory and field observations in other supralittoral amphipods (Steele and Steele 1991). The final model of total abundance (juveniles and adults) retained beach slope in addition to size class and salinity (Fig. 2E–G) as explanatory variables. The retention of beach slope only in the total abundance model remarks the effects of beach morphodynamics on juveniles. The steep slopes in microtidal reflective beaches prevent unpredictable water intrusions in the supralittoral zone due to storms and winds, protecting juveniles that are incapable of escaping towards the dunes (Gómez and Defeo 1999; Defeo and Gómez 2005).

**Figure 3:**
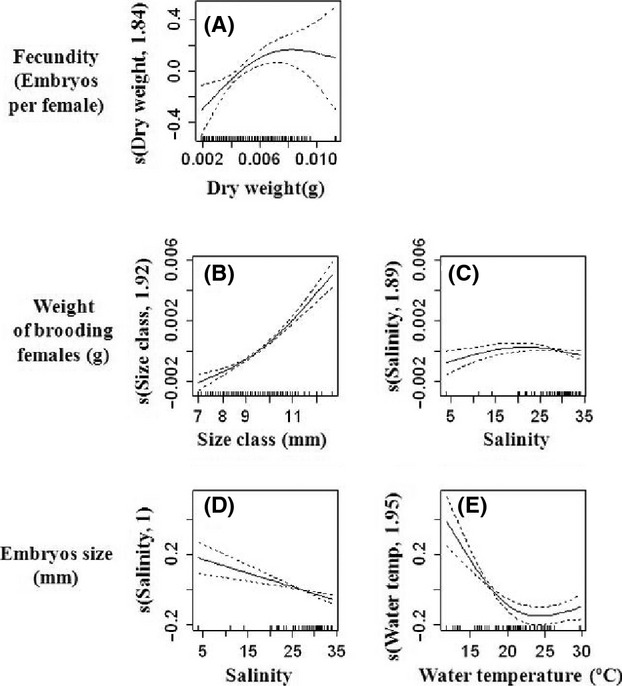
Poisson GAMM for the number of embryos per female (A), and Gaussian GAMM for brooding females body dry weight (B, C) and for embryos size (D, E), showing partial effects of retained environmental predictors. The numbers in the *y*-axis labels indicate the estimated degrees of freedom of the smooth curve. Marks on the *x*-axis indicate the measured values of each variable.

The females fecundity model only retained individual weight as the main predictor (Fig. 3A), indicating that environmental conditions do not have a direct effect on the number of embryos produced. The increase in fecundity with individual weight reveals the importance of considering body weight as proxy of the available energy to invest in reproduction. In addition, when the response variable was body weight of brooding females, individual size and salinity were the relevant variables (Fig. 3B, C), showing once again the importance of salinity in shaping the life history of *A. brasiliensis*. Species' preference for salinities close to 15–20 (Gómez and Defeo 2012) suggests that the decrease in dry weight towards fully freshwater conditions may be due to the energy expenditure required to allow an adequate osmoregulation of females. Metabolic rate may increase as a result of osmotic stress inflicted by the decline in salinity, decreasing the efficiency to absorb nutrients and generate reserves (Normant and Lamprecht 2006). This becomes clear if we consider that ionic regulation is a steady state that requires large amounts of energy to be accomplished (Schmidt-Nielsen 1997). Furthermore, GAMM results revealed a shift in the size at which brooding females reach the same weight at low salinities, in comparison with those that inhabit salinities >15. Thus, for a given size, brooding females exhibit lower weights in low-salinity environments (Fig. 3C and Fig. 6).

Embryos size decreased with salinity (Fig. 3D). This, in conjunction with the fact that females have a limited space in their pouches, allow us to consider the existence of a trade-off between embryos size and fecundity, taking into account that heavier females have more embryos in environments with intermediate and high salinities. Thus, two reproductive strategies could exist: (1) low number of large embryos at low salinities; and (2) high number of small embryos at intermediate/high salinities (Fig. 6). One potential explanation is that females that occur in environments where they have an efficient osmoregulation (salinities >15) exhibit higher weight and energy availability, being able to produce more embryos. By contrast, females in environments where they have a large expenditure in osmoregulation (salinities <15) invest their energy in size but not in number of embryos. The production of larger embryos in low-salinity systems allows future juveniles to invest more energy in osmotic control once they become independent from the parental care. An alternative explanation is the osmotic water uptake that embryos could present at low-salinity waters (Moran and McAlister 2009). Moreover, embryos size decreased with temperature (Fig. 3E). *Gammarus duebeni* and *G. insensibilis* also produce bigger embryos during cold months and smaller ones in the summer (Sheader 1983, 1996). The physiological bases for these observations are not understood, but temperature has a well-documented negative correlation with egg size in many marine taxa, both within and among species (Levitan 2000; Moran and McAlister 2009).

**Figure 4:**
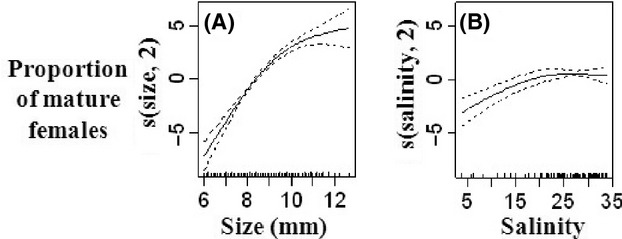
Binomial GAMM (logit scale) of maturity, showing partial effects of retained predictors on mature/nonmature females: (A) size and (B) salinity. The numbers in the *y*-axis labels indicate the estimated degrees of freedom of the smooth curve. Marks on the *x*-axis indicate the measured values of each variable.

Size at maturity in females (Fig. 4A) was affected only by salinity (Fig. 4B). This life-history trait exhibited a shift towards salinities <20. Thus, 50% of females reached maturity at larger sizes or, for a given size, the proportion of mature females decreased in low-salinity environments (Fig. 6). This coincides with the shift caused by salinity in the size at which females reach a certain weight, suggesting a metabolic constraint imposed by osmoregulation in *A. brasiliensis*, and its consequences in body size and maturity of females.

**Figure 5:**
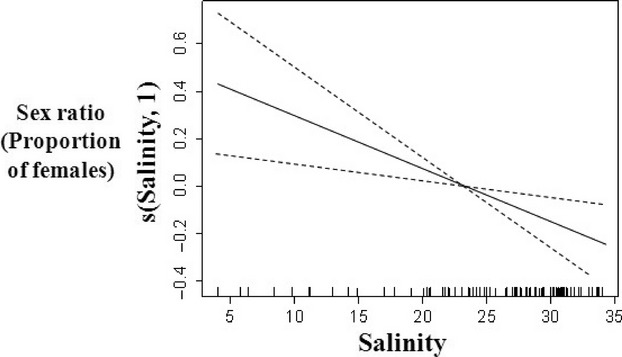
Binomial GAMM (logit scale) of sex ratio (females/males), showing the partial effect of the retained environmental predictor. The standardized mean of the response variable (mean = 0) is equivalent to a prevalence of 0.57 in the original scale of observations. The lowest recorded value was 0.54 (in favour of females) at salinity = 34. The number in the *y*-axis label indicates the estimated degrees of freedom of the smooth curve. Marks on the *x*-axis indicate the measured values of each variable.

**Figure 6:**
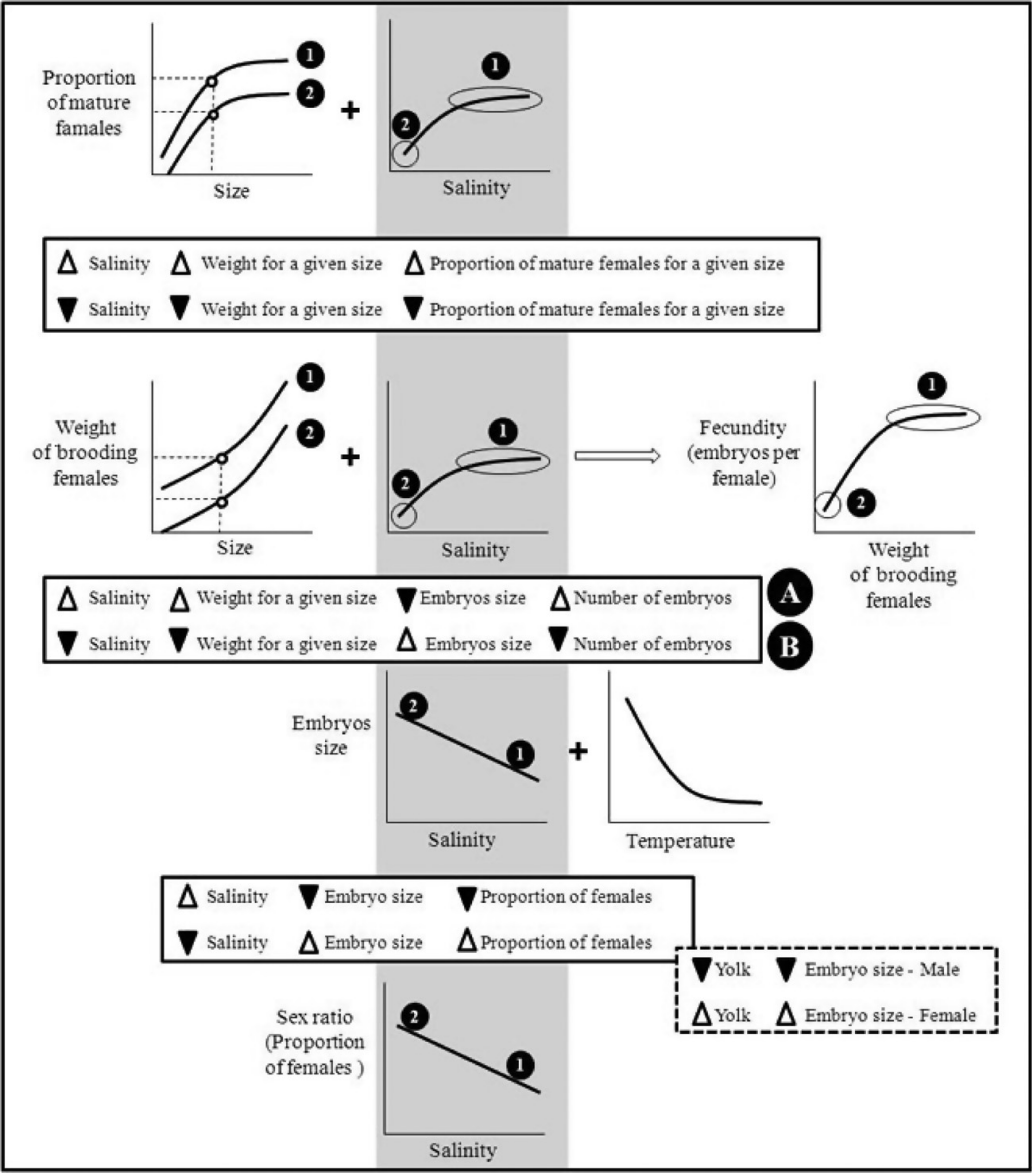
Conceptual diagram of the connection among life-history traits in *Atlantorchestoidea brasiliensis* (see grey shadow). Two environmental scenarios (1 – high salinities, 2 – low salinities) and two reproductive strategies (A – low number of large embryos, B – high number of small embryos) are shown. Doted lines represent a shift in life-history traits for the salinity scenarios based on the additive nature of the models (see “+”). Boxes integrate the information between trait models for both salinity scenarios. The doted box indicates one of the possible explanations for sex determination (see details in text).

Environmental sex determination (ESD) was described in several animal groups (West et al. 2002; Kato et al. 2011; Gamble and Zarkower 2012). In peracarids such as talitrid amphipods, the effects of environmental factors on sex ratio have been documented (Johnson et al. 2001). We found a prevalence of females (Fig. 5), which could be attributed to the local photoperiod (10–14 h of daylight). Studies in *G. duebeni* showed a female prevalence at photoperiods shorter than 14 hours (Bulnheim 1991). The prevalence of females decreased at higher salinities (Fig. 5). Three possible explanations can account for this pattern: (1) the infection of feminizing microsporidean protozoan parasites on embryos of *G. duebeni* populations caused a bias in favour of females, but its incidence is suppressed by salinity levels up to 25–30 (Bulnheim 1991); (2) Epigenetic factors like salinity could stimulate gonad maturity acting directly on the crustaceans' endocrine system, potentially determining the sexual identity of the organisms (Nagaraju 2011); and (3) Yolk allocation could determine the sex of the offspring as in other zoological groups (Radder et al. 2009). In this sense, our results found coincident trends for sex ratio and embryo size in relation to salinity. Although little is known about the mechanisms related to ESD (Kato et al. 2011), the models obtained for *A. brasiliensis* suggest that larger embryos (found at low salinities) could produce females, while smaller embryos (found at high salinities) produce males (Fig. 6).

## Conclusions

Salinity was identified as the only variable that affects all life-history traits of *Atlantorchestoidea brasiliensis*. Low-salinity values demand large amounts of energy for osmoregulation that cannot be assimilated as reserves (body weight) for reproductive costs. This explains why at low-salinity scenarios the individual weight of brooding females and the proportion of mature females were lower for a given size. In addition, salinity affected embryo size and fecundity, the latter through body weight. Low salinities represent a risk to offspring, being necessary to provide energy reserves (yolk) to each embryo to increase their probabilities to survive. Under this adverse scenario, the increase in embryo size and the decrease in female fecundity (due to the limited space in its pouch) represent a trade-off. Furthermore, yolk allocation could explain why large embryos produce females, determining the high prevalence of this sex at low salinities, which should compensate low individual fecundities. Our results highlighted that variables acting on physiology influence the energy balance and determine interconnected responses of life-history traits mainly associated with reproduction, having consequences at the population level. In addition, other variables related to environmental unpredictability (in our case beach slope) can have significant effects on fitness through organisms' survival. In summary, this study revealed that field data of multiple traits and modelling approaches that incorporate random factors are useful to unveil environmental drivers that define concerted responses of life-history traits, providing insights into understand the potential underlying mechanisms.
